# Clinical Prediction Rules for In-Hospital Mortality Outcome in Melioidosis Patients

**DOI:** 10.3390/tropicalmed9070146

**Published:** 2024-06-28

**Authors:** Sunee Chayangsu, Chusana Suankratay, Apichat Tantraworasin, Jiraporn Khorana

**Affiliations:** 1 Department of Internal Medicine, Surin Hospital, Surin 32000, Thailand; chayangsu.sunee@gmail.com; 2 Department of Internal Medicine, Faculty of Medicine, The King Chulalongkorn Memorial Hospital, Chulalongkorn University, Bangkok 10330, Thailand; csuankratay@gmail.com; 3 Clinical Surgical Research Center, Department of Surgery, Faculty of Medicine, Chiang Mai University, Chiang Mai 50200, Thailand; apichat.t@cmu.ac.th; 4 Center of Clinical Epidemiology and Clinical Statistic, Faculty of Medicine, Chiang Mai University, Chiang Mai 50200, Thailand; 5 Division of Pediatric Surgery, Department of Surgery, Faculty of Medicine, Chiang Mai University Hospital, Chiang Mai 50200, Thailand

**Keywords:** melioidosis, mortality rate, *Burkholderia pseudomallei*, sepsis, outcome, clinical prediction rules

## Abstract

Background: Melioidosis, a disease induced by *Burkholderia pseudomallei*, poses a significant health threat in tropical areas where it is endemic. Despite the availability of effective treatments, mortality rates remain notably elevated. Many risk factors are associated with mortality. This study aims to develop a scoring system for predicting the in-hospital mortality from melioidosis using readily available clinical data. Methods: The data were collected from Surin Hospital, Surin, Thailand, during the period from April 2014 to March 2017. We included patients aged 15 years and above who had cultures that tested positive for *Burkholderia pseudomallei*. The clinical prediction rules were developed using significant risk factors from the multivariable analysis. Results: A total of 282 patients with melioidosis were included in this study. In the final analysis model, 251 patients were used for identifying the significant risk factors of in-hospital fatal melioidosis. Five factors were identified and used for developing the clinical prediction rules, and the factors were as follows: qSOFA ≥ 2 (odds ratio [OR] = 2.39, *p*= 0.025), abnormal chest X-ray findings (OR = 5.86, *p* < 0.001), creatinine ≥ 1.5 mg/dL (OR = 2.80, *p* = 0.004), aspartate aminotransferase ≥50 U/L (OR = 4.032, *p* < 0.001), and bicarbonate ≤ 20 mEq/L (OR = 2.96, *p* = 0.002). The prediction scores ranged from 0 to 7. Patients with high scores (4–7) exhibited a significantly elevated mortality rate exceeding 65.0% (likelihood ratio [LR+] 2.18, *p* < 0.001) compared to the low-risk group (scores 0–3) with a lower mortality rate (LR + 0.18, *p* < 0.001). The area under the receiver operating characteristic curve (AUC) was 0.84, indicating good model performance. Conclusions: This study presents a simple scoring system based on easily obtainable clinical parameters to predict in-hospital mortality in melioidosis patients. This tool may facilitate the early identification of high-risk patients who could benefit from more aggressive treatment strategies, potentially improving clinical decision-making and patient outcomes.

## 1. Introduction

Melioidosis is an infectious disease caused by *Burkholderia pseudomallei*. The disease was first described in 1912 [1], but it remains a major public health problem in many parts of the world, particularly in Southeast Asia and Northern Australia [2]. It is found in soil and water in tropical and subtropical regions and can be transmitted to humans through contact with contaminated soil or water, or inhalation of aerosolized droplets. Melioidosis can present with a wide range of clinical features, making it difficult to diagnose. This pathogen has the capability to affect various parts of the body, including the lungs, liver, spleen, kidneys, bones, joints, skin, soft tissue, and multiple other organs [3]. Data from numerous studies consistently indicate a high mortality rate associated with melioidosis. Estimates suggest this rate ranges from 30% to 50% [4,5,6,7,8,9]. This is due to several factors, including the difficulty of diagnosing the disease, the lack of effective treatment options, and the high prevalence of comorbid conditions in patients with melioidosis. Despite the challenges, significant progress in melioidosis research in recent years has led to a better understanding of the epidemiology, pathogenesis, and treatment of the disease. However, there is still much that we do not know about melioidosis, and further research is needed to improve the outcomes for patients. In our study, effectively predicting fatal melioidosis proved difficult despite the use of suitable pharmacotherapy or aggressive treatment. There was no specific therapeutic regimen to treat melioidosis. This study aimed to create a scoring system to predict in-hospital mortality of melioidosis, incorporating factors essential for aggressive treatment strategies.

## 2. Materials and Methods

This retrospective cohort study was conducted at Surin Hospital. Data were collected from existing medical records and documented in a standardized case record form. Informed consent was waived as the research did not involve direct patient interaction and relied solely on pre-existing records. Approval for the study protocol (80/2020) was obtained from the Institutional Ethics Committee.

### 2.1. Participants

The data were collected from patients with melioidosis in Surin Hospital who were admitted from April 2014 to March 2017. Confirmation of melioidosis by all clinical specimen cultures grew *Burkholderia pseudomallei*. In the reviewed chart, it was found that incomplete data or other diagnoses were identified, leading to the exclusion of these patients from the study.

### 2.2. Outcomes

The participants were diagnosed with melioidosis with clinical specimens that grew *Burkholderia pseudomallei*, and after receiving standard treatment, the patients were divided into two groups: the non-survival group and the survival group.

### 2.3. Predictors

In our previous study [10], mortality was found to be influenced by various factors, including age, respiratory rate, abnormal chest X-ray findings, and bicarbonate (HCO_3_) levels, and these factors were used to develop a predictive scoring system [10]. Other parameters collected in this study were categorized into general information, clinical presentation, and laboratory findings.

The general information consisted of age, gender, comorbidity, body mass index (BMI), and duration of illness. Elderly individuals were defined as those aged 60 years or older. Comorbidities included diabetes mellitus (DM), chronic kidney disease (CKD), cirrhosis, thalassemia, heart disease, and cerebrovascular accident (CVA), among others.

The clinical presentation included signs, symptoms, and clinical syndrome. Localized infection was identified as an infection confined to a single site or primary bacteremia without a focal lesion. Bacteremia was defined as positive growth in blood culture without the involvement of a specific organ. Disseminated infection, on the other hand, was characterized by infections occurring at multiple sites whether it involved more than one site infection with or without bacteremia or a single site infection accompanied by bacteremia. Signs and symptoms were used to calculate the Quick Sepsis-related Organ Failure (qSOFA) score, incorporating altered consciousness, respiratory rate, and systolic blood pressure. Additionally, Systemic Inflammatory Response Syndrome (SIRS) criteria were employed.

The laboratory findings included radiologic findings, white blood cell counts with platelet count, blood urea nitrogen (BUN), creatinine (Cr), liver function test, and bicarbonate (HCO_3_) levels. A neutrophil percentage greater than 85 indicated leukocytosis, a platelet count less than 100,000/mm^3^ indicated thrombocytopenia, BUN levels above 20 mg/dL indicated uremia, Cr levels above 1.5 mg/dL indicated renal injury, aspartate aminotransferase (AST) levels above 50 U/L indicated hepatic injury, albumin levels below 3 g/dL indicated hypoalbuminemia, and HCO_3_ levels below 20 mEq/L indicated acidosis. These thresholds denote severe illness in patients. Abnormalities on chest X-rays were identified as infiltrations, which included patchy areas, consolidations, reticular patterns, ground-glass opacities, and cavities.

### 2.4. Statistical Analysis

Statistical analysis utilized STATA version 16.0. Continuous variables were expressed as mean (standard deviation) or median (interquartile range), and categorical variables as counts and percentages. Student’s *t*-test or Mann–Whitney U test and Fisher’s exact test were used as needed. Logistic regression analysis identified factors associated with in-hospital mortality, reported as odds ratios (ORs) with 95% confidence intervals (CI). Significant clinical parameters were selected for model development using a stepwise method. A scoring system was derived from regression coefficients, predicting mortality probability with a cut-off score of 4. Model performance was assessed by positive predictive value (PPV), likelihood ratio (LR), area under the receiver operating characteristic (AuROC) curve, Hosmer–Lemeshow goodness of fit statistics, and calibration plot. Internal validation was conducted using bootstrapping with 200 replicates, and a complete case analysis was applied to handle missing data. The logistic regression analysis included 252 patients to develop the clinical prediction score.

## 3. Results

**Figure 1:**
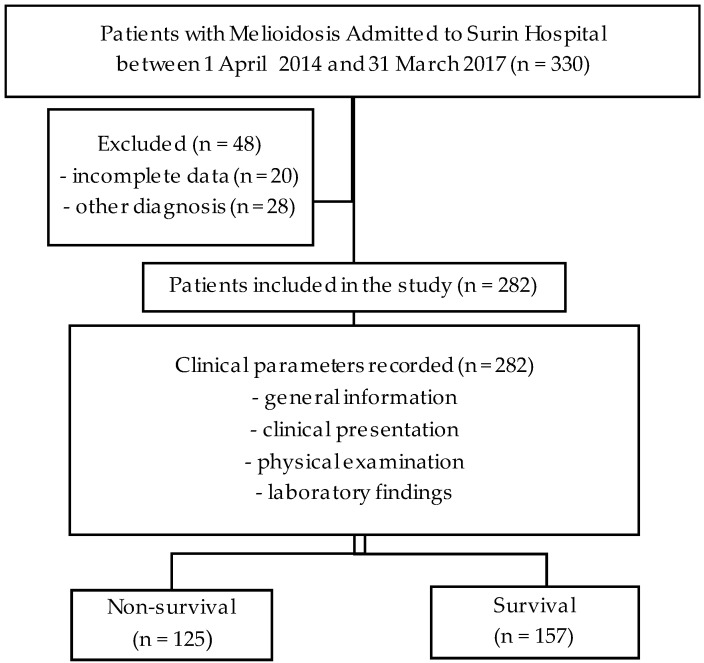
Study flow diagram.

First, data on 330 participants with melioidosis were collected. Then, 282 participants were included in the final analysis, as shown in Figure 1. A total of 125 patients (44.3%) were classified into the non-survival group and 157 patients (55.7%) into the survival group. Both groups had a higher number of males. Most patients were over 50 years old. Common underlying diseases include diabetes mellitus, chronic kidney disease, cirrhosis, and thalassemia. At least one disease was found in both groups. The attached Supplementary Tables S1 and S2 includes detailed information on the sites of infection and the antibiotic regimens used in this study. Further details are provided in Table 1, highlighting the parameters that demonstrated univariable statistical significance for in-hospital mortality of melioidosis, which were age, body mass index, qSOFA score, SIRS score, clinical syndromes, abnormal chest X-ray findings, percentage of neutrophils, platelet count, BUN, Cr, AST, albumin, and HCO_3_.

**Figure 2:**
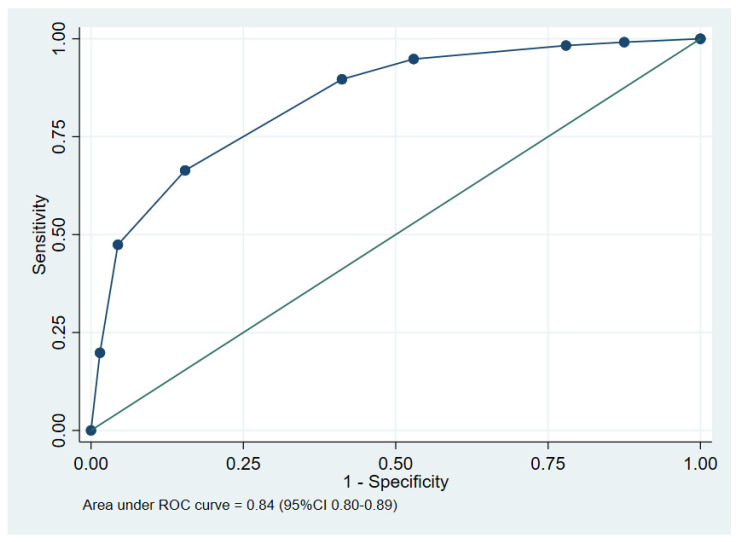
The performance of the clinical prediction score, showing the area under the receiver operating characteristic (ROC) curve.

Multivariable logistic regression was carried out. The parameters selected in the model as defined in the method are shown in Table 2. The final model included a qSOFA score ≥ 2, abnormal chest X-ray findings, serum creatinine ≥ 1.5 mg/dL, AST ≥ 50 U/L, and HCO_3_ ≤ 20 mEq/L. The scoring system for predicting in-hospital mortality in melioidosis patients, based on derived coefficients, is detailed in Table 3. The scores, ranging from 0 to 7 points, categorized patients into two risk groups with a cut-off of 4 points, as shown in Table 4. Scores of 0 to 3 points indicated the low-risk group, while scores of 4 to 7 points identified the high-risk group. The receiver operating characteristic (ROC) curve and the performance of the clinical prediction score model are illustrated in Figure 2.

**Figure 3:**
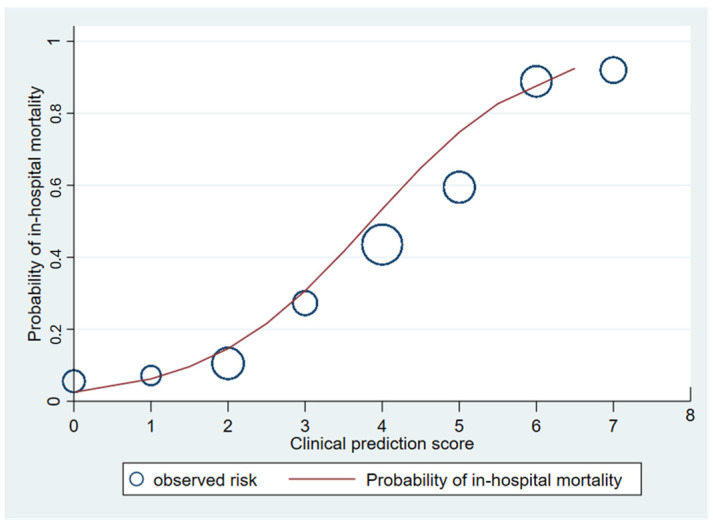
Observed risk (circle) versus clinical prediction score (solid line) of in-hospital mortality of melioidosis.

The area under the receiver operating characteristic (AuROC) curve of the clinical score model demonstrated a prediction affinity of 0.84 (95% CI 0.80–0.89). The Hosmer–Lemeshow test indicated a good model fit with no evidence of a lack of fit (*p* = 0.480). The clinical score model was fit for the prediction of in-hospital mortality in melioidosis patients in this dataset. The calibration of the model is shown in Figure 3, which compares the observed risk with the clinical prediction score of in-hospital mortality. The bootstrapping method was performed for internal validation, which showed a consistent AuROC of 0.84 (95% CI 0.79–0.89), with the model optimism at 0.006 (ranging from −0.312 to 0.367), and the bootstrap shrinkage was 0.940 (95% CI 0.67–1.18).

## 4. Discussion

Melioidosis is a common disease that has been known for a long time. However, the mortality rate remains high even though there have been effective treatments available for a long time. Both ceftazidime and meropenem are effective treatments, with no significant difference in outcomes [11,12,13,14]. However, meropenem is recommended for severe cases [3]. Therefore, if it is possible to predict which melioidosis cases are at high risk of severe disease and high mortality, it may be possible to change the treatment regimen early or provide more intensive treatment. For this reason, we aspired to find clinical prediction rules for fatal melioidosis. In some cases, melioidosis is treated with meropenem or combined with trimethoprim–sulfamethoxazole (TMP-SMX) because it is thought that a broader spectrum of antibiotics will be more effective. However, this may lead to the overuse of antibiotics, which may not be necessary in all cases. The results of this study showed that patients with a score of more than 4 have a high risk of mortality.

The results of this retrospective study of 282 patients with melioidosis demonstrate that a qSOFA of ≥ 2, abnormal chest X-ray findings, creatinine ≥ 1.5 mg/dL, aspartate aminotransferase ≥ 50 U/L, and bicarbonate ≤ 20 mEq/L served as indicators for in-hospital mortality prediction. These parameters, available at the time of admission including vital signs and lab findings, were integrated into a prediction model. These parameters were incorporated into a composite scoring system, assigning points to each factor. The total score ranged from 0 to 7. Patients with a high score (4–7) had a significantly higher mortality rate exceeding 65.0% (likelihood ratio [LR+] of 2.18, *p* < 0.001). Conversely, a low score (0–3) predicted a substantially lower mortality rate (LR + 0.18, *p* < 0.001). The model demonstrated good test performance with an ROC (receiver operating characteristic) curve exceeding 0.80, indicating a strong ability to discriminate between survivors and non-survivors.

Previous studies [15] have identified pneumonia, age at diagnosis, blood urea nitrogen (BUN) level, blood bilirubin level, lymphocyte count, and blood bicarbonate level as predictors of mortality from melioidosis, with higher scores associated with increased mortality rates. Our clinical score model incorporates additional clinical features derived from symptoms and vital signs that are incorporated into the qSOFA score. An elevated qSOFA score indicates the presence of sepsis or potential organ dysfunction in the patient. Abnormal chest X-ray findings, suggestive of pneumonia or severe illness, have also been identified as a prognostic factor in multiple studies. In some cases, these findings are used as a key factor in deciding to extend the duration of intravenous antimicrobial therapy to reduce mortality and recurrence rates [16,17,18]. Increased creatinine levels signified potential kidney dysfunction, a risk factor for worse outcomes. Increased AST levels may indicate liver damage, another marker of severe illness. Low bicarbonate level suggests metabolic acidosis, a complication associated with critical illness. These findings are consistent with previous studies [8,15] and support the importance of these factors in predicting mortality from melioidosis.

In addition to clinical symptoms and routine laboratory test results, which are used in clinical prediction models, several studies [19,20] have found that plasma cytokine concentrations can also affect mortality in melioidosis patients. These cytokines include IL-6, IL-8, IL-10, and TNF-α. A recent study [21] used IL-6 and IL-8 levels to create a clinical prediction model that was combined with existing clinical data. The model that included the biomarkers was able to predict 28-day mortality in melioidosis patients more accurately than the model that used clinical data alone. The model was also validated in an internal validation set and an external validation set. The results confirmed that the model that included the biomarkers was more effective at predicting mortality than the model that used clinical data alone. However, measuring plasma cytokine concentrations is not easily feasible and cannot be routinely performed in clinical practice, which presents a significant challenge for hospitals with limited resources. Future studies should focus on making this test more accessible and affordable.

This study’s scoring system was derived from a relatively small sample size and validated within the same dataset. External validation on a separate cohort is crucial to assess the model’s performance in diverse clinical settings. Ensuring the model’s effectiveness across various environments requires thorough external evaluation.

## 5. Conclusions

Our study identified five independent predictors of fatal melioidosis: qSOFA score ≥ 2, abnormal chest X-ray findings, creatinine ≥ 1.5 mg/dL, AST ≥ 50 U/L, and HCO_3_ ≤ 20 mEq/L. Based on these factors, we developed a scoring system to predict in-hospital mortality from melioidosis. This scoring system can be a valuable tool for clinicians to identify high-risk patients who may benefit from early admission to the intensive care unit, more aggressive supportive care, more intensive monitoring to detect complications promptly, and earlier initiation of powerful antibiotics, such as meropenem or combination therapy with TMP-SMX.

## Figures and Tables

**Table 1:** Baseline characteristics of non-surviving and surviving melioidosis patients.

Characteristics	Missing *n* (%)	Non-Survival (*n* = 125)	Survival (*n* = 157)	*p*-Value
Male ^1^, *n* (%)	0	89 (71.2)	118 (75.2)	0.499
Age (years) ^2^, mean ± SD	0	59.0 ± 15.5	55.0 ± 13.2	0.022
Age ≥ 60 years ^1^, *n* (%)		68 (54.4)	62 (39.5)	0.016
Comorbid at least 1 disease ^1^, *n* (%)	0	100 (80.0)	127 (80.9)	1.881
Comorbid ^1^, *n* (%) (DM/CKD/Cirrhosis/Thalassemia)	0	75 (60.0)	100 (63.7)	0.539
qSOFA score ^3^, median (IQR)	0	1 (1–2)	1 (0–1)	<0.001
qSOFA score ≥ 2 ^1^, *n* (%)		49 (39.2)	17 (10.8)	<0.001
SIRS score ^2^, mean ± SD	2 (0.7)	2.9 ± 0.9	2.5 ± 1.1	0.002
SIRS score ≥ 2 ^1^, *n* (%)		115 (92.0)	125 (80.7)	0.009
Body mass index ^2^, mean ± SD	19 (6.7)	20.5 ± 3.7	21.5 ± 3.8	0.025
Clinical syndromes ^1^, *n* (%)	0			<0.001
Localized or bacteremia *		35 (28.0)	80 (51.0)	
Disseminated form **		90 (72.0)	77 (49.0)	
Abnormal chest X-ray finding ^1^, *n* (%)	2 (0.7)	106 (85.5)	71 (45.5)	<0.001
%Neutrophils ^2^, mean ± SD	3 (1.1)	85.5 ± 11.2	81.6 ± 10.7	0.003
%Neutrophils ≥ 851, *n* (%)		81 (64.8)	61 (39.6)	<0.001
Platelet, cells/mm^3 3^, median (IQR)	2 (0.7)	163,000 (84,000–219,000)	213,000 (151,000–323,000)	<0.001
Platelet < 100,000 cells/mm^3 1^, *n* (%)		37 (29.6)	16 (10.3)	<0.001
BUN, mg/dL^3^, median (IQR)	3 (1.1)	37 (22–59)	17 (12–28)	<0.001
BUN ≥ 20 mg/dL ^1^, *n* (%)		97 (77.6)	61 (39.6)	<0.001
Creatinine, mg/dL ^3^, median (IQR)	4 (1.4)	2.1 (1.2–3.4)	1.1 (0.8–1.6)	<0.001
Creatinine ≥ 1.5 mg/dL^1^, *n* (%)		79 (63.7)	44 (28.6)	<0.001
AST, U/L ^3^, median (IQR)	24 (8.5)	109 (61–261)	55 (36–98.5)	<0.001
AST ≥ 50 U/L ^1^, *n* (%)		101 (85.6)	78 (55.7)	<0.001
Albumin, g/dL ^2^, mean ± SD	23 (8.2)	2.5 ± 0.6	2.8 ± 0.6	<0.001
Albumin ≤ 3 g/dL ^1^, *n* (%)		97 (81.5)	85 (60.7)	<0.001
HCO_3_, mEq/L ^2^, mean ± SD	5 (1.8)	17.3 ± 6.5	22.1 ± 5.6	<0.001
HCO_3_ ≤ 20 mEq/L ^1^, *n* (%)		86 (69.4)	42 (27.5)	<0.001

DM = diabetic mellitus; CKD = chronic kidney disease; qSOFA = Quick Sepsis-related Organ Failure Assessment; SIRS = Systemic Inflammatory Response Syndrome; BUN = blood urea nitrogen; AST = aspartate aminotransferase; HCO_3_ = bicarbonate; SD = standard deviation; IQR = interquartile range; * localized form—infection confined to a single site or primary bacteremia without a focal lesion; bacteremia—positive growth in blood culture without the involvement of a specific organ; ** disseminated form—infections involving multiple sites can include either more than one site infection with or without bacteremia or a single site infection accompanied by bacteremia. ^1^ reported as a count or percentage and Fisher’s exact test was used for analysis. ^2^ reported as mean ± standard deviation and Student’s *t*-test was used for analysis. ^3^ reported as median and interquartile range and Kruskal–Wallis was used for analysis.

**Table 2:** Multivariable odds ratio, and 95% CI of selected predictive parameters derived from logistic regression after multiple imputation.

Parameters	Univariable OR	95% CI of uOR	*p*-Value	Multivariable OR	95% CI of mOR	*p*-Value
qSOFA score ≥ 2	5.31	2.86–9.85	<0.001	2.39	1.12–5.11	0.025
Abnormal Chest X-ray	7.05	3.91–12.73	<0.001	5.86	2.79–12.29	<0.001
Creatinine ≥ 1.5 mg/dL	4.39	2.65–7.28	<0.001	2.80	1.38–5.69	0.004
AST ≥ 50 U/L	4.72	2.56–8.71	<0.001	4.03	1.93–8.42	<0.001
HCO_3_ ≤ 20 mEq/L	5.98	3.55–10.07	<0.001	2.96	1.48–5.92	0.002

qSOFA = Quick Sepsis-related Organ Failure Assessment; AST = aspartate aminotransferase; HCO_3_ = bicarbonate; OR = odds ratio; uOR = univariable odds ratio; mOR = multivariable odds ratio.

**Table 3:** Item scoring scheme for predictive parameters for In-Hospital fatal melioidosis based on coefficients of selected indicators.

Diagnostic Parameter	Coefficients	Transformed Coefficient	Assigned Score
qSOFA ≥ 2			
No	-	-	0
Yes	0.87	1	1
Abnormal Chest X-ray			
No	-	-	0
Yes	1.77	2.03	2
Creatinine ≥ 1.5 mg/dL			
No	-	-	0
Yes	1.03	1.18	1
AST ≥ 50 U/L			
No	-	-	0
Yes	1.39	1.60	2
HCO_3_ ≤ 20 mEq/L			
No	-	-	0
Yes	1.08	1.25	1

**Table 4:** Distribution of risk of in-hospital fatal melioidosis, LR+, and 95% CI of LR+.

Risk Level	Fetal Melioidosis (%)	Non-Fatal Melioidosis (%)	PPV (%)	LR+	95% CI of LR+	*p*-Value
Low (score 0–3)	12 (13.0)	80 (87.0)	13.0	0.18	0.10–0.31	<0.001
High (score 4–7)	104 (65.0)	56 (35.0)	65.0	2.18	1.76–2.69	<0.001

## Data Availability

The datasets utilized in the current study can be obtained from the corresponding author upon reasonable request.

## Supplementary Materials

The following supporting information can be downloaded at: https://www.mdpi.com/article/10.3390/tropicalmed9070146/s1, Table S1: Organ involvement in melioidosis (a single patient may have multiple organs affected); Table S2: Antibiotic-based treatment.
