# Supplementary material for: Clinical Prediction Rules for In-Hospital Mortality Outcome in Melioidosis Patients
Source: Trop Med Infect Dis. 2024 Jun 28;9(7):146. doi: 10.3390/tropicalmed9070146 (PMC11281151; doi:10.3390/tropicalmed9070146)
Supplement: Supplementary file 1 [file tropicalmed-09-00146-s001.zip › tropicalmed-3017549-supplementary.pdf]

Tropical Medicine and Infectious Disease

Table S1. Organ involvement in melioidosis (a single patient may have multiple organs affected).

Organ Involved	Number of Patients (*n* = 282)
Pulmonary melioidosis	170 (60.3%)
Splenic abscess	37 (13.1%)
Hepatic abscess	25 (8.9%)
Urinary tract (prostate/renal)	22 (7.8%)
Skeletal melioidosis (bone/joint)	28 (9.9%)
Skin and soft tissue (abscess or cellulitis)	47 (16.7%)

Table S2. Antibiotic-based treatment.

Antibiotics	Number of Patients (*n* = 282)
Ceftazidime	164 (58.2%)
Carbapenem (meropenem/imipenem)	64 (22.7%)
Amoxycillin/clavulanic acid	7 (2.5%)
Others	47 (16.7%)
